# Glycemic Control Promotes Pancreatic Beta-Cell Regeneration in Streptozotocin-Induced Diabetic Mice

**DOI:** 10.1371/journal.pone.0008749

**Published:** 2010-01-18

**Authors:** Eric J. Grossman, David D. Lee, Jing Tao, Raphael A. Wilson, Soo-Young Park, Graeme I. Bell, Anita S. Chong

**Affiliations:** 1 Department of Surgery, The University of Chicago, Chicago, Illinois, United States of America; 2 Department of Medicine, The University of Chicago, Chicago, Illinois, United States of America; 3 Department of General Surgery, Rush Medical Center, Chicago, Illinois, United States of America; University of Bremen, Germany

## Abstract

**Background:**

Pancreatic beta-cells proliferate following administration of the beta-cell toxin streptozotocin. Defining the conditions that promote beta-cell proliferation could benefit patients with diabetes. We have investigated the effect of insulin treatment on pancreatic beta-cell regeneration in streptozotocin-induced diabetic mice, and, in addition, report on a new approach to quantify beta-cell regeneration *in vivo*.

**Methodology/Principal Findings:**

Streptozotocin-induced diabetic were treated with either syngeneic islets transplanted under the kidney capsule or subcutaneous insulin implants. After either 60 or 120 days of insulin treatment, the islet transplant or insulin implant were removed and blood glucose levels monitored for 30 days. The results showed that both islet transplants and insulin implants restored normoglycemia in the 60 and 120 day treated animals. However, only the 120-day islet and insulin implant groups maintained euglycemia (<200 mg/dl) following discontinuation of insulin treatment. The beta-cell was significantly increased in all the 120 day insulin-treated groups (insulin implant, 0.69±0.23 mg; and islet transplant, 0.91±0.23 mg) compared non-diabetic control mice (1.54±0.25 mg). We also show that we can use bioluminescent imaging to monitor beta-cell regeneration in living MIP-*luc* transgenic mice.

**Conclusions/Significance:**

The results show that insulin treatment can promote beta-cell regeneration. Moreover, the extent of restoration of beta-cell function and mass depend on the length of treatment period and overall level of glycemic control with better control being associated with improved recovery. Finally, real-time bioluminescent imaging can be used to monitor beta-cell recovery in living MIP-*luc* transgenic mice.

## Introduction

Insulin-secreting pancreatic beta-cells proliferate in response to increasing demand for insulin and also after physiological injury [1], [2], [3], [4], [5], [6], [7], [8]. It is generally accepted that beta-cells have a finite life span and that dying beta-cells are continuously replaced [3], [9], [10], [11], [12]. This notion raises the possibility of enhancing base-line replication of beta-cells as a therapeutic approach for the treatment of diabetes patients with type 1 or type 2 diabetes. Indeed, there are clinical case-reports of beta-cell regeneration enabling the complete recovery from type 1 diabetes [13]. However, in the majority of patients, the reported level of recovery is not sufficient to cure, or even maintain glucose homeostasis [7], [8]. A better understanding of the physiological conditions and molecular mechanisms regulating beta-cell proliferation and/or regeneration are necessary in order for beta-cell replication-based therapies to become a reality.

We recently described functional beta-cell regeneration in a mouse model of streptozotocin (STZ)-induced diabetes [14]. Here, we extend these observations by investigating the effects of insulin treatment and glycemic control on beta-cell regeneration in this model. Current methods for quantifying beta-cell regeneration require the sacrifice of mice and analysis of pancreatic sections, thus precluding the real-time measurement of beta-cell regeneration. As a consequence, the kinetics of beta-cell regeneration are poorly understood. We report a new approach that allows studies of beta-cell regeneration in living mice utilizing bioluminescent imaging and MIP-*luc* transgenic mice [15].

## Materials and Methods

### Mice

C57BL/6 mice obtained NCI (Frederick, MD) or Jackson Laboratory (Bar Harbor, ME) were used as donors and recipients of kidney subcapsular islet transplants. CD1 transgenic mice expressing firefly (*Photinus pyralis*) luciferase (*luc*) under the control of the mouse insulin I promoter (MIP- *luc*) [15] were used as recipients of kidney subcapsular islet transplantation. 9–12 week old female mice were made diabetic by a single intraperitoneal (IP) injection of STZ (150 mg/kg, Sigma Chemical, St. Louis, MO). Diabetic mice with non-fasted blood glucose values >400 mg/dl for more than 2 consecutive days (SureStep; Lifescan, Milpitas, CA) were used as recipients of islet grafts or insulin implants. All studies were performed in accordance to protocols approved by the University of Chicago Institutional Animal Care and Use Committee.

### Islet Isolation and Transplantation

Syngeneic islets from C57BL/6 mice were isolated following intraductal collagenase digestion (Collagenase P, 0.3 mg/ml; Roche, Indianapolis, IN) and purification by Ficoll gradient centrifugation (Sigma, St. Louis, MO) as previously described [14], [16]. Approximately 200 islets were transplanted under the kidney capsule.

### Exogneous Insulin Implants

Insulin was administered via subcutaneous ‘LinBit Implants for Mice’ (LinShin, Toronto, ON, Canada). The implants were titrated for blood glucose levels of <250 mg/dl.

### Blood Glucose Monitoring

Random non-fasted blood glucose levels were determined three times weekly from the tail vein using a SureStep glucometer.

### Bioluminescent Imaging

Bioluminescent optical imaging was performed using a Xenogen IVIS 200 imaging system (Xenogen, Alameda, CA) as previously described [15]. Briefly, MIP-*luc* mice were fasted for 4 h, shaved, then anesthetized with isofluorane (using the Xenogen system). Mice were placed on their sides on the imaging stage and an overlay image was initially taken. Mice were then injected IP with 15 mg/ml D-luciferin in sterile PBS (150 mg/kg) and exactly 14 min after injection of D-luciferin, a bioluminescent image was captured utilizing an exposure time of 1 minute. Subsequent image processing including quantification of bioluminescence was conducted using Living Image Software v. 2.05 (Xenogen).

### Histological Studies

Beta-mass was determined as described previously [14]. Briefly, the pancreas was removed, weighed, fixed, embedded and then serially step-sectioned. Every 10th section was stained with anti-insulin polyclonal antibody (Zymed, South San Francisco, CA; ∼10 sections per mouse) and images of each section were captured on a Zeiss Axiovert 200 M microscope. The insulin positive and total pancreas area were quantified with Image J (National Institutes of Health, Bethesda, MD; http://rsb.info.nih.gov/ij/), and the relative ratio of insulin positive areas to the entire pancreas area was determined. The beta-cell mass was calculated by multiplying the relative ratio by the total weight of the pancreas.

### Intraperitoneal Glucose Tolerance Test

An intraperitoneal glucose tolerance test (IPGTT) was performed as previously described [14]. Briefly, after four hours of fasting, the mice received an intraperitoneal injection of dextrose (2 g/kg), and blood glucose levels determined from the tail vein at 30-minute intervals.

### Statistics

Data are presented as means±SEM and evaluated for statistical significance by ANOVA (SuperANOVA v. 1.11; Abacus Concepts, Berkeley, CA). A *P* value of <0.05 was considered to be significant.

## Results

### Effect of Insulin Treatment on Beta-Cell Function in STZ-Diabetic Mice

The experimental design to study beta-cell regeneration was divided into two phases: treatment and monitoring.

#### Treatment Phase

STZ-induced C57BL/6 diabetic female mice, with non-fasted blood glucose values >400 mg/dl for more than 2 consecutive days, were randomly assigned to the islet transplant or insulin implant group. Mice (N = 10 per group) were then monitored for either 60 or 120 days with three times weekly blood glucose measurements.

#### Monitoring Phase

After either 60 or 120 days, the islet transplant was removed by nephrectomy and the insulin implant removed. Blood glucose monitoring was continued for an additional 30 days to allow for the assessment of endogenous beta-cell function. At the end of this 30 day monitoring phase (i.e. 90 or 150 days after induction of diabetes with STZ), the mice were sacrificed and histology performed to quantify beta-cell mass.

**Figure 1:**
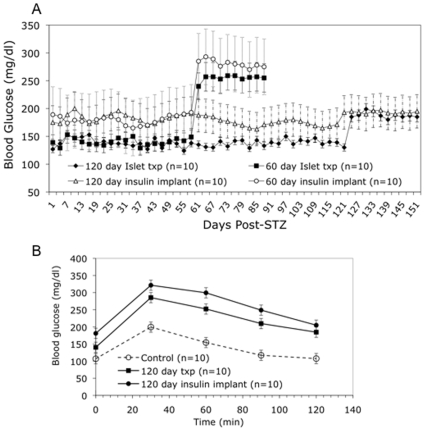
Glycemic Control in Streptozotocin-induced Diabetic Mice. A. Non-fasted (random) blood glucose throughout the treatment and monitoring phases. B. IPGTT comparing glucose tolerance in 120-day insulin-treated STZ-diabetic mice and non-diabetic controls. Txp – transplant.

Both islet transplantation and insulin treatment were effective at preventing hypergylcemia and recurrence of diabetes (Table 1 and Fig. 1A), with blood glucose control by islet transplantation superior to insulin implants (139.4±7.5 and 181.6±8.3 mg/dl respectively, P<0.05). Following the removal of the transplanted islets or discontinuation of insulin therapy, all treatment groups showed a significant (P<0.05) increase in blood glucose levels. Both the 60-day islet transplant and implant groups redeveloped diabetic glucose levels during the Monitoring Phase (255.3±25. and 280.9±35.8 mg/dl, respectively). In contrast, the 120-day islet transplant and insulin implant groups maintained stable non-diabetic blood glucose levels (188.6±31.7 and 194.2±27.3 mg/dl, respectively). At the end of the 30-day monitoring phase, mice from both 120-day groups underwent an IPGTT (Fig. 1B). Both groups showed an abnormal glucose profile compared to non-diabetic controls.

**Table 1:** Blood glucose levels (mg/dl) in STZ-diabetic mice treated with insulin by insulin implant or islet transplant under the kidney capsule.

	60-day insulin implant	60-day islet transplant	120-day insulin implant	120-day islet transplant
Treatment Phase	183.3±18.8	141.4±26.3	171.8±22.7	143.4±18.7
Monitoring Phase	280.9±35.8	255.3±25.2	194.2±27.3	188.6±31.7

### Effect of Insulin Treatment on Beta-Cell Mass in STZ-Diabetic Mice

**Figure 2:**
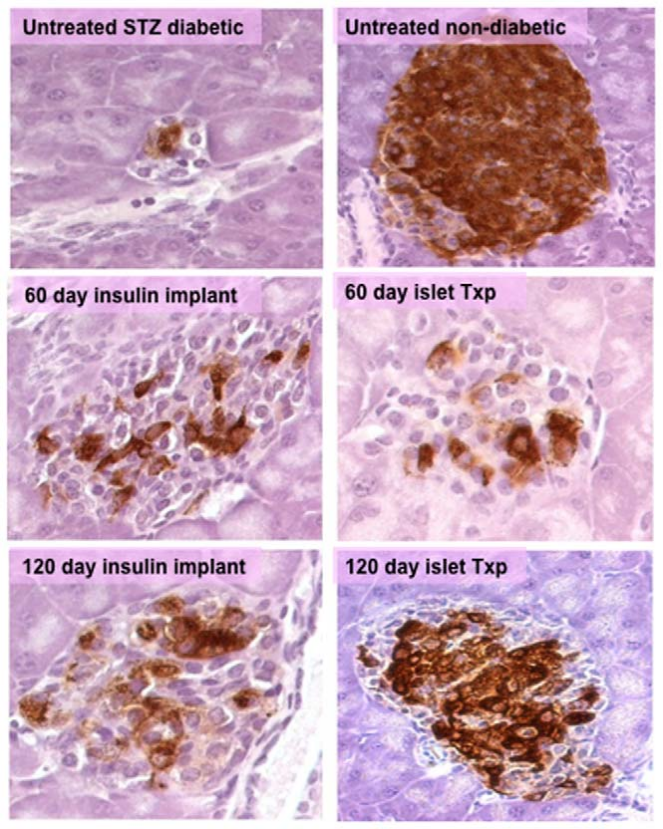
Representative pancreatic islets showing insulin staining (brown) in untreated STZ-diabetic mice, non-diabetic control and STZ-diabetic mice treated with insulin implants or islet transplant (txp) under the kidney capsule for 60 or 120 days (40x).

There was no significant increase in beta-cell mass in the 60-day-insulin-treated groups compared to STZ-treated controls (untreated STZ-control group, 0.09±0.01 mg; islet transplant, 0.14±0.09 mg; and insulin implant, 0.18±0.13 mg) (Table 2). In contrast, there was a significant increase in beta-cell mass in both the 120-day-insulin-treated groups compared to untreated STZ-induced diabetic mice (islet transplant, 0.91±0.23 mg; insulin implant, 0.69±0.22 mg). The maximum beta-cell regeneration occurred in the 120-day islet transplant group achieving a 60% recovery of beta-cell mass that was statistically greater than the 45% recovery in 120-day insulin implant group (P<0.05). The islets from the 120-day islet transplant group were large and densely populated with insulin-producing beta-cells as compared to the other treatment groups (Fig. 2).

**Table 2:** Beta-cell mass (mg) in STZ-diabetic mice treated with insulin by insulin implant or islet transplant under the kidney capsule.

	Non-diabetic control	Untreated STZ- diabetic mice	60-day insulin implant	60-day islet transplant	120-day insulin implant	120-day islet transplant
Beta-cell mass (mg)	1.52±0.25	0.09±0.01	0.18±0.13	0.14±0.09	0.69±0.22*	0.91±0.23*

Untreated STZ-diabetic B6 mice were examined 4–8 days after STZ-induced diabetes, which when untreated results in severe diabetes and death.

* P<0.05 compared to untreated STZ-diabetic mice.

### Real-Time Quantification of Beta-Cell Recovery

Traditional measures of beta-cell mass preclude real-time quantification and thus the kinetics of beta-cell regeneration cannot be accurately ascertained. Our data suggest that significant beta-cell regeneration occurred in the 60–120 day post-STZ treatment period, but we were not able to define the kinetics of regeneration within this 60 day period. We therefore tested whether biweekly bioluminescence imaging could successfully be used to quantify beta-cell mass following destruction and regeneration. Because bioluminescence emission is a dynamical chemical reaction dependent on luciferin availability, numerous optimization experiments were initially performed to determine the time point (14 minutes post luciferin injection) at which maximal bioluminescence emission could be captured. All bioluminescence measurements were then performed in precisely the same manner to allow for results at different time points to be compared.

**Figure 3:**
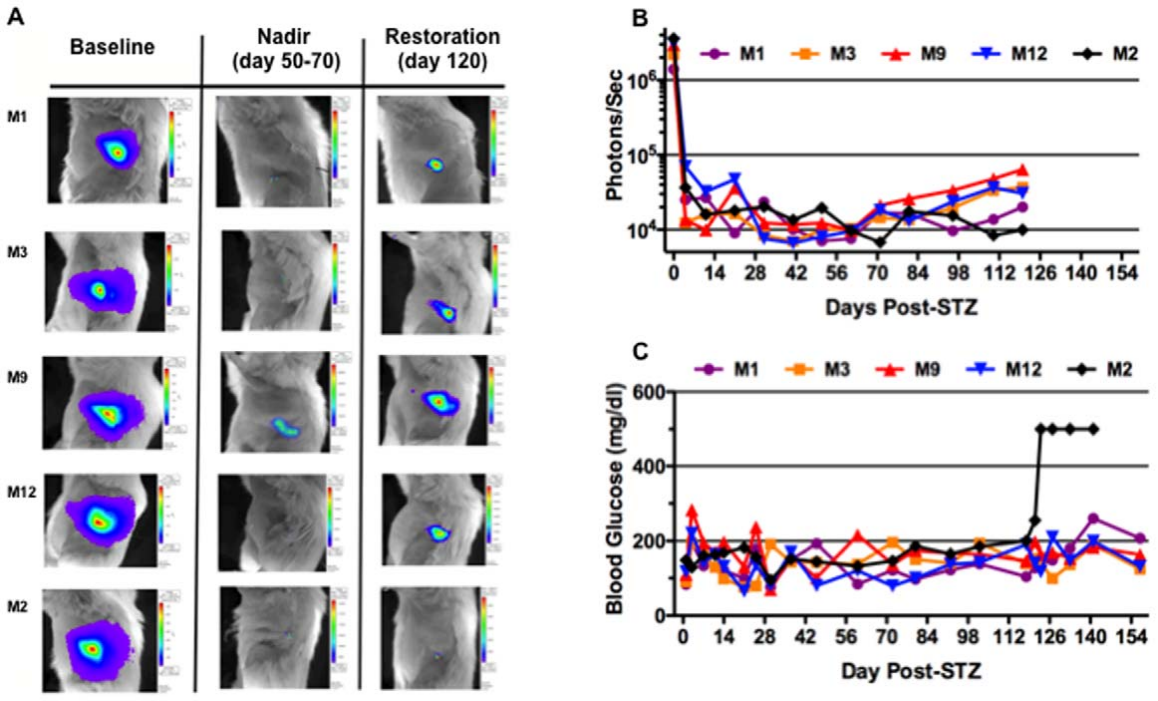
Bioluminescence and Glycemic Control in Streptozotocin-induced Diabetic MIP-*luc* Mice. A. Representative bioluminescent images of five MIP-*luc* transgenic mice made diabetic with STZ and insulin treated by islet transplant under the kidney capsule. B. Bioluminescent signal (photons/sec) during the treatment phase. C. Blood glucose levels during the treatment (120 days) phase.

STZ-induced diabetic MIP-*luc* female mice (C57BL/6 background) were treated with 200 syngeneic wild-type C57BL/6 islets placed under the kidney capsule. The transplanted islets do not express the MIP-*luc* transgene, and consequently, the bioluminescent signal reflects the endogenous beta-cell mass. The transplanted islets were removed at 120 days via nephrectomy beta-cell function monitored for an additional 30 days. The baseline bioluminescent signal was similar for all five MIP-*luc* mice. After STZ-induced diabetes, bioluminescence decreased continued to do so, reaching a nadir approximately 60 days after the induction of diabetes in all five mice (Fig. 3). After 60 days post-STZ treatment phase, four of the five mice (M1, M3, M9 and M12) showed a persistent increase in bioluminescent signal. In contrast, M2 demonstrated a transient increase in bioluminescent that eventually was lost. At 120 days post-STZ treatment, the bioluminescent signal of M1, M3, M9, and M12 showed a 5.2±1.5 fold increase compared to their nadir while M2, did not.

All five MIP- *luc* mice maintained normal random blood glucose levels (148.3±26 mg/dl) prior to nephrectomy, indicating appropriate function of the transplanted islets. However, following nephrectomy, M1, M3, M9 and M12 were able to maintain glycemic control (180±47, 136±30, 166±20, and 152±37 mg/dl, respectively), indicative of functional recovery of the native beta-cells. In contrast, M2, demonstrated severe hyperglycemia (500 mg/dl) following nephrectomy, consistent with the lack of recovery of bioluminescent signal at 120-day post-STZ treatment (Figure 3C; 0.28% of pre-STZ levels). Of note is that although M1, M3, M9 and M12 maintained normal glycemic control following nephrectomy, the animals with the largest bioluminescent signal at 120 days post-STZ treatment (M3, M9 and M12) maintained the tightest glycemic control while mouse M1, which had the lowest signal, had the poorest glycemic control. Thus, relatively small differences in bioluminescence between individual mice appear to be associated with dramatic differences in glycemic control, highlighting the sensitivity of this experimental approach to monitor beta-cell mass regeneration *in vivo*.

## Discussion

Recent reports of functional pancreatic beta-cell regeneration in murine models [17], [18] has generated much excitement and controversy [3], [19]. Despite the increasingly accepted notion that pancreatic beta-cells have the ability to regenerate, either from remaining beta-cells or existing beta-cell precursors, and to restore euglycemia after the induction of diabetes in laboratory rodent models, the understanding and clinical relevance of beta-cell regeneration is still incomplete. In particular, observations that beta-cell regeneration derives primarily from existing beta-cells and that insulin secreting beta-cells remain functional in patients with established type 1 diabetes, raise the possibility that beta-cell regeneration may contribute to the recovery in patients with autoimmune diabetes [20], [21], [22], [23]. The results of the studies described here suggest that insulin treatment by implants or islet transplantation promotes beta-cell regeneration in the STZ-diabetic mouse model of beta-cell regeneration, and does so in a time dependent manner with longer treatment periods associated with greater recovery. They also suggest that the overall degree control of glycemic control affects regeneration with better control accelerating recovery of beta-cell mass and function. Further studies are needed to determine the optimal length of time of insulin treatment for complete recovery of beta-cell mass and function in this model.

Studies of beta-cell regeneration in the MIP-*luc* transgenic mouse model may provide additional insight into the process of beta-cell regeneration by facilitating sequential real-time measurements of beta-cell mass in living animals [15], [24], [25], [26]. Following STZ-treatment bioluminescence continued decline to a nadir at day 60, followed by a spontaneous increase in endogenous beta-cell bioluminescence from day 60–120 post-STZ. Notably small differences in recovery of bioluminescence (0.28–2.13% of pre-STZ bioluminescence levels) were associated with dramatic differences in the control of blood glucose. Also the restoration of beta cell mass measured by bioluminescence at day 0–120 post-STZ was quantitatively lower than that determined by conventional morphometric analysis, which was performed at 90 or 150 days post-STZ treatment. The different times at which beta-cell mass was determined and the differential sensitivity of these techniques under low beta-cell mass conditions could have contributed to these differences. More extensive measurements of beta cell mass under the two conditions are necessary to clarify the bases for these differences in perceived regeneration as determined by the two experimental approaches. Despite these differences, only the bioluminescence approach can permit the quantification of changes in beta-cell mass in an individual mouse over time, which we believe can provide valuable insights into the kinetics of beta-cell regeneration that the more conventional approach cannot.

In conclusion, our studies of beta-cell regeneration in the STZ-diabetic mouse may have implications for patients with type 1 diabetes and suggest that if the ongoing immunological destruction of beta-cells can be prevented, proliferation of the remaining beta-cells in the presence of insulin (and possibly other agents) may lead to a restoration of normal glucose tolerance.
